# Characterization of an active ingredient made of nanoscale iron(oxyhydr)oxide for the treatment of hyperphosphatemia†

**DOI:** 10.1039/d1ra00050k

**Published:** 2021-05-14

**Authors:** Magdalena Bäumler, Sebastian P. Schwaminger, Daniela von der Haar-Leistl, Simon J. Schaper, Peter Müller-Buschbaum, Friedrich E. Wagner, Sonja Berensmeier

**Affiliations:** Bioseparation Engineering Group, Department of Mechanical Engineering, Technical University of Munich Boltzmannstraße 15 85748 Garching Germany s.berensmeier@tum.de; Fraunhofer Institute for Process Engineering and Packaging (IVV), Department of Process Development for Plant Raw Materials Giggenhauser Str. 35 85354 Freising Germany; Functional Materials Group, Departement of Physics, Technical University of Munich James-Franck-Straße 1 85748 Garching Germany; Heinz Maier-Leibnitz Zentrum (MLZ), Technical University of Munich Lichtenbergstr. 1 85748 Garching Germany; Experimental Astro-Particle Physics Group, Departement of Physics, Technical University of Munich James-Franck-Straße 1 85748 Garching Germany

## Abstract

Kidney disease is one of the main non-communicable diseases. Every year millions of people worldwide die from kidney dysfunction. One cause is disturbances in the mineral metabolism, such as abnormally high phosphate concentrations in the blood, medically referred to as hyperphosphatemia. A new active ingredient based on nanoscale iron(oxyhydr)oxide with particle sizes below 3 nm surrounded by an organic coating has been developed for a more effective treatment. The examination of the structural properties of these particles within this study promises to gain further insights into this improved effectiveness. More than half of the active ingredient consists of organic substances, the rest is mostly iron(oxyhydr)oxide. Analyzes by transmission electron microscopy (TEM), small-angle X-ray scattering (SAXS), and dynamic light scattering (DLS) show that the organic molecules act as stabilizers and lead to ultrasmall iron(oxyhydr)oxide cores with a size of 1.0–2.8 nm. The nanoparticles coated with the organic molecules have an average size of 11.7 nm. At 4.2 K, the nanoparticles display a magnetic hyperfine field of 45.5 T in the Mössbauer spectrum, which is unusually low for iron(oxyhydr)oxide. The material is also not ferrimagnetic. Combining these results and taking into account the composition of the nanoparticles, we identify low crystalline ferrihydrite as the most likely phase in the iron(oxyhydr)oxide nuclei. At the same time, we want to emphasize that a final identification of the crystal structure in iron(oxyhydr)oxides can be impeded by ultrasmall particle sizes. In summary, by a combinatorial characterization, we are able to observe extraordinary properties of the ultrasmall nanomaterial, which is the basis for the investigation of the high phosphate-binding efficacy of this active ingredient.

## Abstract 2

The combination of different analytical methods, supported by TEM, DLS, SAXS, Mössbauer spectroscopy, and SQUID, allows more accurate characterization of a new nanoscale active ingredient based on iron(oxyhydr)oxide against hyperphosphatemia.

## Graphical abstract

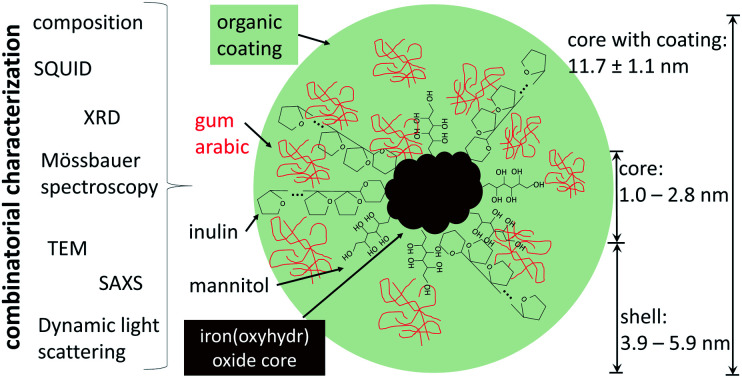

## Introduction

Kidney diseases are among the most important non-communicable diseases. The US Department of Health and Human Service estimated that about 15% of the American population, often unconsciously, suffers from chronic kidney disease.^1^ In 2017, more people died of kidney dysfunction in the United States than of breast or prostate cancer.^2^ Moreover, the Global Burden of Disease (GBD) estimated that in 2015 1.2 million people worldwide died from a decreased renal function.^3^ The high morbidity and mortality of hemodialysis patients are known to be strongly associated with disorders of mineral metabolism like hyperphosphatemia.^4^ A reduced renal function, for example, often leads to an increase of the phosphate level in the blood (>1.46 mmol L^−1^), which is medically referred to as hyperhosphatemia.^5^ To reduce the phosphate concentration in blood in the long term, medical treatment with phosphate adsorbing substances is the most efficient therapeutic approach.^6^ These oral drugs react with phosphate in the gastrointestinal tract, reducing the intestinal absorption of dietary phosphate.^7^

In this work, we aim to improve the treatment of hyperphosphatemia by expanding the knowledge of a novel and potentially highly effective active ingredient recently presented by Wagner *et al.*^8^ Nguyen *et al.* showed that a precursor of this substance has a higher phosphate binding capacity in a simulated gastrointestinal passage compared to the most commonly prescribed sevelamer carbonate and sucroferric oxyhydroxide.^9,10^ In addition, the effectiveness is less affected by the pH variation in the gastrointestinal tract.^9,10^ Like the sucroferric oxyhydroxide, the substance is also based on iron(oxyhydr)oxides. However, the two substances differ in particular in the type of organic components present in the active ingredients. While starch and sucrose are the main components of the sucroferric oxyhydroxide, Wagner *et al.* use inulin, mannitol, and gum arabic in the synthesis.^8,11^ The organic components can have different functions in the substances. Organic molecules are known to interact with iron(oxyhydr)oxides by binding on the surfaces *via* ligand exchange reactions and/or electrostatic attraction forces.^12^ This adsorption effects, for example, the particle size during the synthesis as well as during storage by influencing the surface charge of the particles. When only a small number of organic molecules covers the particles, this can lead to a decrease of the positive surface charge of the iron(oxyhydr)oxides and aggregation of the nanoparticles may occur.^12^ A high concentration of organic matter, in turn, leads to the building of an organic layer on iron(oxyhydr)oxides which can cause electrostatic repulsion between the nanoparticles.^12^ In consequence, aggregation and coagulation growth are hampered and the particle size is decreased.^12^ Thus, Bachhar *et al.* observed smaller particle sizes when magnetite was coated with polyacrylic acid or dextran in comparison to uncoated nanoparticles.^13^ Eusterhues *et al.* reported a decrease in particle size for ferrihydrite in presence of organic matter in comparison to organic-free ferrihydrite.^14^

In addition, organic molecules can influence the crystal arrangement in iron(oxyhydr)oxides. In general, about 16 different iron(oxyhydr)oxide species are distinguished in terms of their crystal arrangement and their composition.^15^ The presence of organic molecules can affect the crystal properties, as they can lead to the formation of different structures during synthesis, impair or prevent structural transformation, and can also reduce the degree of crystallinity.^15^

The effects of the organic components on the particle size, the crystal structure, and thus on the composition and surface groups will in turn change the phosphate-binding of the material. Phosphate is mainly adsorbed on the surface of the iron(oxyhydr)oxides adsorbent by the formation of inner-sphere complexes and ligand exchange reactions.^16,17^ Properties like type and number of chemical groups or area of the adsorbent surface have a significant impact on the adsorption process. The particle composition, the crystal structure, and its degree of crystallinity are also important. These factors can also depend on each other. In summary, the knowledge of adsorbent composition and properties is indispensable for explaining phosphate adsorption processes on iron(oxyhydr)oxide-based materials. In consequence, a structural analysis of these materials is important to understand how phosphate adsorbs on their surfaces.

Nguyen *et al.* postulate in their report that the extremely high phosphate binding capacity is due to the high organic content in the nano-material.^10^ Within this study, we want to expand the knowledge about this novel active ingredient and want to clarify the following questions:

(1) What is the composition of this highly effective active ingredient?

(2) What is the distribution of the components in the nanomaterial and what is the structural arrangement?

(3) How do the iron(oxyhydr)oxide structures, which have a strong affinity for phosphate binding, and the organic components, which influence the iron(oxyhydr)oxide cores, interact to build this high effective phosphate-binding substance?

However, it is commonly known that different strengths and limitations can complicate the choice of suitable analytical methods for nanomaterials. We, therefore, follow a combinatorial characterization approach as proposed by Mourdikoudis *et al.*^18^ We determine the composition of the material in detail and use transmission electron microscopy (TEM), dynamic light scattering (DLS), and small-angle X-ray scattering (SAXS) to investigate the size distribution of the active substance. This knowledge will provide crucial information on the distribution of the components and the adsorptive surface in the active ingredient. Finally, measurements by Mössbauer spectroscopy and a superconducting quantum interference device (SQUID) allow conclusions to be drawn about the structure of the contained iron(oxyhydr)oxide part.

Based on this knowledge, we improve the knowledge about the novel active substance and the effectiveness of a future drug.

## Experimental

### Synthesis

We synthesized the iron(oxyhydr)oxide-based nanoparticles by using a co-precipitation method according to the protocol described by Wagner *et al.* in example 1.^8^ We solubilized 7.55 g iron(iii) chloride hexahydrate (FeCl_3_·6H_2_O, Sigma-Aldrich, ≥99%) and 3.2 g iron(ii) chloride tetrahydrate (FeCl_2_·4H_2_O, Honeywell, ≥99%) in 50 mL purified water (18.2 MΩ cm^−1^). Another solution contained 5 g inulin (inulin HT, Spinrad) and 15 g d-mannitol (Sigma-Aldrich, ≥99%) in 100 mL of a 1.5 M sodium hydroxide solution. The temperature of both solutions was kept at 4 °C. For precipitation, the iron salt solution was added to the sodium hydroxide solution as quickly as possible under vigorous stirring with a magnetic stirrer. After 15 minutes of further vigorous stirring, we added 3 mL hydrogen peroxide solution (30 wt% in H_2_O, Honeywell, puriss). The suspension was stirred for further 5 minutes at 4 °C, before being heated to 60 °C and stirring for further 15 minutes after this temperature was reached. In order to remove the water-soluble sodium and chloride ions and unbound inulin and mannitol, we cleaned the nanoparticle suspension using a dialysis membrane (ZelluTrans T1, 3.5 kDa, Roth) in a beaker filled with 3 L of purified water. The dialysate was changed three times a day for three days. Afterward, we separated larger particles by centrifuging the suspension at 3900 g for 10 minutes and discarding the sediment. We dissolved 3 g gum arabic (from acacia tree, Sigma) in the supernatant resulting in an iron to gum arabic ratio of approximately 0.56. After concentrating in a rotary evaporator (Rotavapor from Büchi Labortechnik, Swiss) at 100 mbar and 60 °C for 30 minutes, the suspension was freeze-dried using a freeze dryer Beta 1-8 LMC-2 from Martin Christ Gefriertrocknungsanlagen, Germany, and crushed with a spoon.

### Investigation of the nanoparticle composition

Sample preparation for the investigation of the nanoparticle composition was performed at least threefold per sample.

#### Fe quantification

The quantification of total Fe in the nanoparticles was performed with a 1,10-phenanthroline colorimetric assay adapted to a method from Nguyen.^9^ The phenanthroline reagent solution contained 1 g L^−1^ 1,10-phenanthroline hydrochloride monohydrate (Alfa Aesar, ≥99%), 14 mL L^−1^ acetic acid (≥99.5%), and 21.7 g L^−1^ sodium acetate trihydrate (Chemsolute, ≥99.5%) in purified water. For sample preparation, 60 to 120 mg of the nanoparticles was solubilized with 2.5 mL hydrochloric acid in a 50 mL volumetric flask, which was filled to the mark with purified water. 1 mL of this solution was then diluted to the mark in a 25 mL volumetric flask with purified water. For the measurement, 140 μL of hydroxylamine hydrochloride solution (100 g L^−1^, Bernd Kraft, analytical grade) and then 980 μL of the 1,10-phenanthroline reagent solution were added to 280 μL of the final dilute sample solution. We measured the absorbance at 510 nm within 30 minutes in a universal microplate spectrophotometer μQuant (BioTek Instruments, VT, USA). Dilutions of an iron standard solution (1000 mg L^−1^ in 0.5 M nitric acid, Merck) with concentrations between 5 and 15 mg L^−1^ were measured for calibration.

#### Fe^2+^ quantification

120 to 200 mg of the nanoparticles was dissolved in a 25 mL polypropylene volumetric flask with 0.5 mL dilute sulfuric acid (50%, v/v). We immediately added 0.5 mL of a 5 mol L^−1^ solution of ammonium fluoride (Fluka, analytical grade) before diluting the sample to the mark with purified water. For the measurement, we added 900 μL 1,10-phenanthroline reagent solution as well as 100 μL of the ammonium fluoride solution to 100 μL of the sample solution and measured the absorbance at 510 nm using a microplate spectrophotometer after 10–30 minutes. The amount of Fe^2+^ in the samples was quantified using Fe^2+^ standard solutions. For this purpose, dilute solutions of an iron standard solution (1000 mg L^−1^ in 0.5 M nitric acid, Merck) with concentrations between 5 mg L^−1^ and 40 mg L^−1^ and 240 mL L^−1^ hydroxylamine hydrochloride were prepared. The standard solutions were heated to 40 °C for 60 minutes. They were measured in the same way as the sample solutions.

#### Mannitol and inulin quantification

The d-mannitol and inulin content was measured by high-performance anion exchange chromatography (HPAEC) using a Dionex ICS-3000 Ion Chromatography System equipped with a pulsed amperometric detector. The analytes were separated at 25 °C using a CarboPac PA10 column and a CarboPac PA10 guard column (Thermo Fisher Scientific) with a flow rate of 0.25 mL min^−1^. Ultrapure water (A), 150 mM NaOH (B), and 1 M sodium acetate with 150 mM NaOH (C) were used as eluents with the following gradient: 0–25 min, 10% B, 0% C; 30–35 min 100% C, 40–55 min 10% B, 0% C. Due to a lack of standards for quantification of fructooligosaccharides, the inulin was hydrolyzed prior to analysis.^19^ For this purpose, 50 mg nanoparticles was dissolved in 1.5 mL 2% hydrochloric acid and incubated for 60 minutes at 80 °C. Then, 30 μL was diluted with 0.1 mM sodium hydroxide in a 10 mL volumetric flask. 500 μL of this sample solution was further dissolved with 1.5 mL of a standard solution containing 5 g sodium azide, 10 mg d-fructose, 10 mg d-glucose as well as 10 mg maltitol (as internal standard) in 500 mL of purified water. Sample solutions were filtered with a 0.2 μm nylon syringe filter (Berrytec GmbH, Germany) before analysis.

The inulin content results from the sum of the glucose and fructose content. Since one molecule of water per broken bond is incorporated into the monomers during the hydrolysis of inulin, the measured values obtained still have to be corrected by a factor of 0.9. In addition, preliminary tests have shown that the hydrolysis of inulin can produce other molecules besides glucose and fructose, which are not recorded by this method. For the specified method, the recovery of inulin is 90% (determined in five separate measurements). These two correction factors cancel each other, wherefore in this case no additional correction is necessary.

#### Chloride quantification

The chloride content was determined photometrically by a mercuric thiocyanate assay. 30 mg of the nanoparticles was dissolved within 10 min at 80 °C with 2 mL dilute nitric acid (3%, v/v). Due to matrix effects that reduce the photometric signal, a standard-addition method was performed for quantification. For this purpose, 40 μL of the prepared sample solution was pipet in four reaction tubes and 0 μL, 1 μL, 2 μL, and 3 μL of a chloride standard solution (Certipur chloride standard, 1000 mg L^−1^ Cl, Merck KGaA, Germany) was added.

The total volume was filled up to 1 mL with purified water. For the measurement, we added 160 μL mercury thiocyanate solution (contained in the chloride test kit for method 8113, Hach Company) and 80 μL ferric ion solution (contained in the chloride test kit for method 8113, Hach Company) and measured the absorbance at 455 nm. For the evaluation, the absorbance of the blank value, which was determined with 40 μL dilute nitric acid (3%, v/v) instead of sample solution, is subtracted from all measured values. A linear regression of the measured values is performed with the concentration of the added chloride standard as *x*-data and the absorbance of the sample solution (corrected by the blank value) as *y*-data. The unknown chloride concentration *c*_Cl_ (mg L^−1^) in the sample solution results from the intersection of the linear line with the *x*-axis and is calculated by eqn (1).1
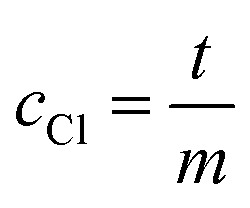
where *t* (−) is the *y*-axis intercept of the regression curve and *m* (L mg^−1^) is the slope of the regression curve.

#### Gum Arabic and sodium quantification

The gum arabic content of the nanoparticles was determined indirectly by quantifying the calcium content in the gum arabic. The sodium and the calcium content were determined using inductively coupled plasma mass spectrometry (ICP-MS) with a 7700 series ICP-MS system (Agilent Technologies, USA). The ICP-MS was equipped with an ASX-520 autosampler (Agilent Technologies, USA) and a micromist nebulizer (Agilent Technologies, USA). The system operated at 1550 W plasma power, 0.3 rps peristaltic pump speed, and 15 L min^−1^ carrier gas flow rate (argon). For sample preparation, 60 to 120 mg of nanoparticles was solubilized in a 50 mL volumetric flask with 5 mL dilute sulfuric acid (50%, v/v) and filled to the mark with dilute nitric acid (3%, v/v). For calibration, a sodium standard solution (sodium standard for IC, 1000 mg L^−1^ Na, Sigma Aldrich, MO, USA) and a calcium standard solution (Certipur calcium standard solution, 1000 mg L^−1^ Ca in 3% v/v HNO_3_, Merck KGaA, Germany) were diluted in 3% v/v nitric acid to concentrations between 1 and 50 mg L^−1^ sodium and 1 and 15 mg L^−1^ calcium. A solution containing 10 mg L^−1^ rhodium (Certipur rhodium ICP standard solution, 1000 mg L^−1^ Rh in 3% v/v HNO_3_, Merck KGaA, Germany) was added as an internal standard with the peristaltic pump during the measurement.

#### Quantification of residual moisture

The residual moisture in the dried material was quantified by Karl Fischer titration with a KF titrator Aqua 40.00 (ECH Elektrochemie Halle GmbH) equipped with a headspace module. 10 to 20 mg of the dried samples was weighed into a headspace vial and sealed with an aluminum crimp cap. To correct for the air humidity, three separate, empty vials were sealed with a crimp cap at the same time as the sample weight. The water content in these vials was subtracted from the measured values as a blank value. For the measurement, the vials were heated to 90 °C in the headspace module and the evaporating water was quantified in the KF titrator. The end-point criterion was the drift stabilization (increase ≤ initial drift + 2 μg min^−1^). All results on the component quantification were subsequently corrected by the residual moisture content according to eqn (2).2
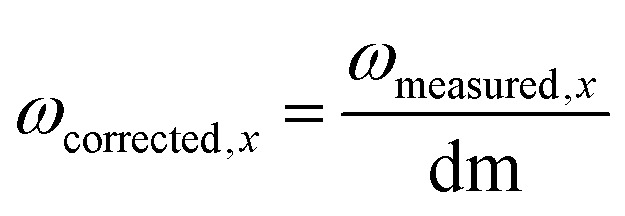
where *ω*_corrected,*x*_ (mg g^−1^) is the content of fraction *x* corrected by the residual moisture, *ω*_measured,*x*_ (mg g^−1^) is the measured content of fraction *x*, and dm (wt%) is the dry matter of the sample.

### Investigation of particle size and shape

Particle size, crystallite size, and particle shape were studied with dynamic light scattering (DLS), transmission electron microscopy (TEM), and small-angle X-ray scattering (SAXS).

#### Dynamic light scattering

A Delsa Nano C Particle Analyzer (Beckman Coulter GmbH, Germany) was used. 10 mg of freeze-dried sample material was dispersed in 10 mL of purified water. Each sample was measured with a 658 nm laser under a 165° scattering angle recording 100 single measurements.

#### Transmission electron microscopy

TEM examination was performed using a JEM-1400(PLUS) 120 kV transmission electron microscope (JEOL GmbH, Japan). For the sample preparation, we used the nanoparticle suspension before freeze-drying and put a few drops of this on a carbon-coated copper grid.

#### Small-angle X-ray scattering

The SAXS data were measured at room temperature with a SAXSLAB GANESHA instrument using a Cu K_α_ X-ray source (XENOCS GeniX3D ULD SL) giving a monochromatic beam with a wavelength *λ* = 1.54 Å. A glass capillary with a high transmission of 67.7% and a low scattering background in the investigated *q*-range was used as a sample holder. A 2D DECTRIS PILATUS 300k detector with a pixel size of 172 × 172 μm^2^ was used. The sample-detector distance of 406.2 mm was chosen to cover a *q*-range from 0.22 nm^−1^ to 7.06 nm^−1^, where the signal of the nanoparticles was expected. To avoid air scattering and obtain a good signal, measurement was conducted for 45 min at a vacuum of 10^−2^ mbar. The obtained 2D detector image was azimuthally integrated and the *q*-range was calculated from the parameters given above. The reduced data (intensity over *q*) were fitted with the program SASfit^20^ using an exponential background for the sample holder and a spherical form factor with a bimodal log-normally distributed radius. The bimodal log-normal distribution (BiLogNorm(*q*, *μ*_1_, *σ*_1_, *μ*_2_, *σ*_2_, *N*_1_, *N*_2_)) used is a linear combination of two log-normal distributions according to eqn (3).

3 BiLogNorm(*q*, *μ*_1_, *μ*_2_, *σ*_1_, *σ*_2_, *N*_1_, *N*_2_) = LogNorm1(*q*, *μ*_1_, *σ*_1_, *N*_1_) + LogNorm2(*q*, *μ*_2_, *σ*_2_, *N*_2_)

Where *q* is the value of the scattering vector, *N*_1,2_ the number of scattering particles, *μ*_1,2_ are the median values, and *σ*_1,2_ are the widths of the log-normal distributions (LogNorm(*q*, *μ*, *σ*, *N*)) according to eqn (4).4
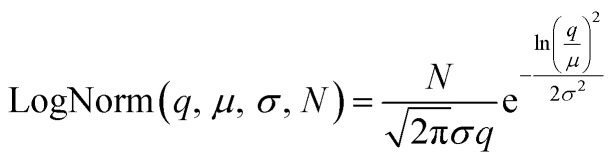


### Crystal structure and structural properties

#### X-ray diffraction

X-ray diffraction (XRD) was performed at room temperature with a Stadi-P (STOE & Cie GmbH, Germany) equipped with a Mo K_α_ source (*λ* = 0.7093 Å), K_α_1__ radiation (*λ* = 0.7093 Å) and a Mythen 1 K detector (DECTRIS Ltd., Switzerland).

#### Mössbauer spectroscopy

Mössbauer spectra were measured in transmission geometry with a standard electromechanical spectrometer (Halder Elektronik, Germany) using a sinusoidal velocity waveform and a source of ^57^Co in rhodium. The spectrometer was calibrated against an α-iron foil at ambient temperature. The 14.4 keV γ-rays were detected with a Kr proportional counter with single-channel analyzer windows set on both, the 14.4 keV photo-peak and the escape peak. For measurements at 4.2 K, the source and the absorber were cooled in a liquid He bath cryostat. The spectra thus obtained were quadrupole doublets at room temperature and magnetic sextets at 4.2 K. In both cases, the spectra are broadened. Therefore, they were least-square fitted with Gaussian distributions of quadrupole splitting for the room temperature spectra and hyperfine fields for the 4.2 K spectra. The resulting Voigt profiles were calculated by superimposing 40 individual doublets or sextets. Their Lorentzian lines were assumed to be the same for all superimposed spectra but were otherwise allowed to vary freely. Further details will be discussed together with the experimental results. Isomer shifts are given with respect to the ^57^Co in the Rh-source, having the same temperature as the absorber. In order to refer them to α-iron at room temperature, 0.11 mm s^−1^ must be added for the room temperature spectra. For the 4.2 K spectra, 0.24 mm s^−1^ have to be added to take the second-order Doppler shift resulting from the different source temperatures into account.

#### Superconducting quantum interference device

The magnetic properties were determined using a superconducting quantum interference device (SQUID) magnetometer MPMS (Quantum Design Inc., CA, USA). Hysteresis curves were measured at 300 K and 4.2 K, and the magnetic field was varied from −50 kOe to +50 kOe. The temperature dependence of the magnetization was measured in zero-field-cooled (ZFC) and field-cooled (FC) mode. First, the sample was cooled from 300 K to 2 K without an externally magnetic field. Thereafter, the ZFC curve was obtained by measuring the magnetization of the sample, while gradually increasing the temperature to 300 K in an applied magnetic field of 200 Oe. The sample was then cooled to 2 K with an external applied field of 200 Oe and the FC curve was determined by measuring the magnetization of the sample while gradually increasing the temperature from 2 K to 300 K.

## Results and discussion

**Fig. 1:**
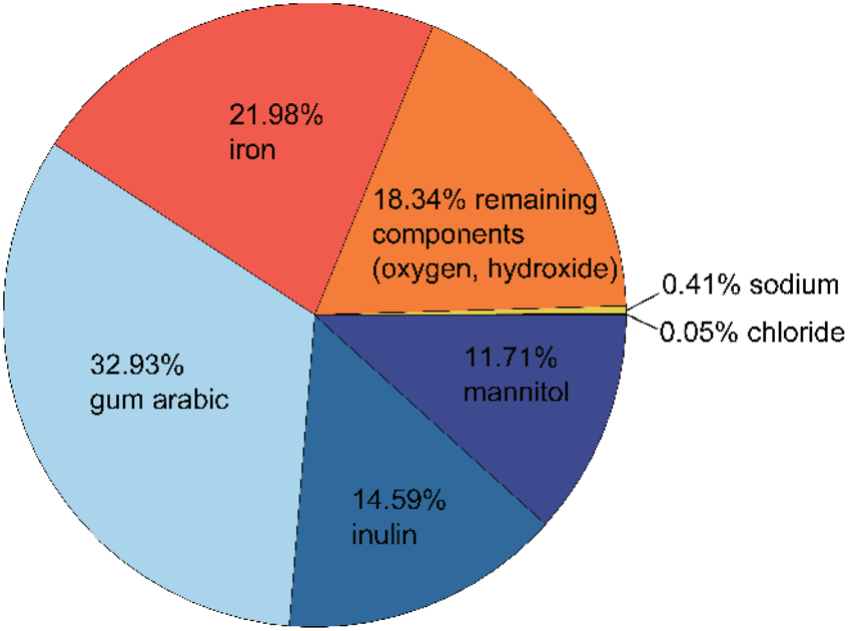
Composition of the active ingredient: organic components are shown in blue, iron(oxyhydr)oxide components in orange, inorganic components in yellow. All data are given as mass fractions, which are corrected for the residual water content according to eqn (2).

As mentioned above, we want to improve the understanding of the mode of action of the novel phosphate-binding substance, for which a better knowledge of the composition and properties of the adsorbent is essential. We start with the particle composition of the active ingredient. Due to the small production scale and as a test for reproducibility, we prepared several samples as described above. The exact composition of all three samples is shown in Table S1 in the ESI.† A graphical summary of the average composition is presented in Fig. 1.

**Fig. 2:**
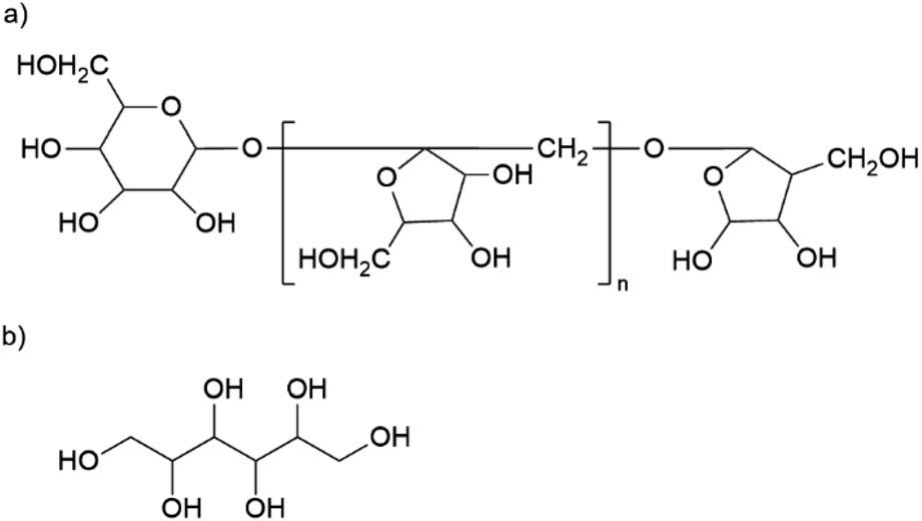
Molecular structure of (a) inulin from chicory root and (b) mannitol.

The material has a high organic content. The largest fraction is gum arabic with an average of 329 mg g^−1^. This exudate of certain acacia trees consists of a widely branched polysaccharide with arabinose, rhamnose, glucuronic acid, and galactose as main monomers. In addition, gum arabic has a small content of protein (<5%) attached to the polysaccharide chain. ^21^ The nanoparticles also contain about 146 mg g^−1^ of inulin, a naturally occurring polysaccharide consisting of fructose with a terminal glucose molecule (*cf.*Fig. 2a). Due to the natural origin, the chain length varies. In this study, we use inulin derived from the chicory root. This has typically a chain length between 2 and 60 units.^22^ The third organic component is mannitol with approximately 117 mg g^−1^. Fig. 2b shows the molecular structure of this sugar alcohol.

The second-largest fraction is iron with approximately 220 mg g^−1^, of which only a small proportion are divalent iron ions (≤3 mg g^−1^). Despite several days of cleaning by dialysis, the nanoparticles still contain residues of sodium (3.6 to 4.4 mg g^−1^) and chloride (0.4–0.5 mg g^−1^), which are remnants of the sodium hydroxide and iron chloride salts used in the synthesis. About 18% of the particle components cannot be assigned to any of the analyzed components. Based on the known educts used in the production process, we assume that this mass fraction of the nanoparticles is mainly oxygen and/or hydroxide ions in the iron(oxyhydr)oxide structures.

Overall, these particles contain a high proportion of organic components in comparison with other synthesized iron(oxyhydr)oxide nanoparticles.^14^ In most cases, the use of organic stabilizers is even completely avoided within the synthesis of iron(oxyhydr)oxides.^16,23,24^ As mentioned above, the presence of organic matter can have different effects on nanoparticle properties. Due to their high surface-to-volume ratio, nanoparticles tend to aggregate in order to reduce surface energy.^25^ In comparable studies, organic molecules such as dextran or starch were often used as stabilizers for iron(oxyhydr)oxide nanoparticles to prevent aggregation during long-term storage.^26^ Adding the substances during the co-precipitation of the nanoparticles can reduce even crystal growth by preventing growth by coagulation.^13^ As a result, the sizes of the iron(oxyhydr)oxide core and the overall particle sizes are reduced in comparison with organic-free nanoparticles. All three organic educts used in our synthesis are known for their stabilizing effects on iron(oxyhydr)oxide nanoparticles.^26–30^

**Fig. 3:**
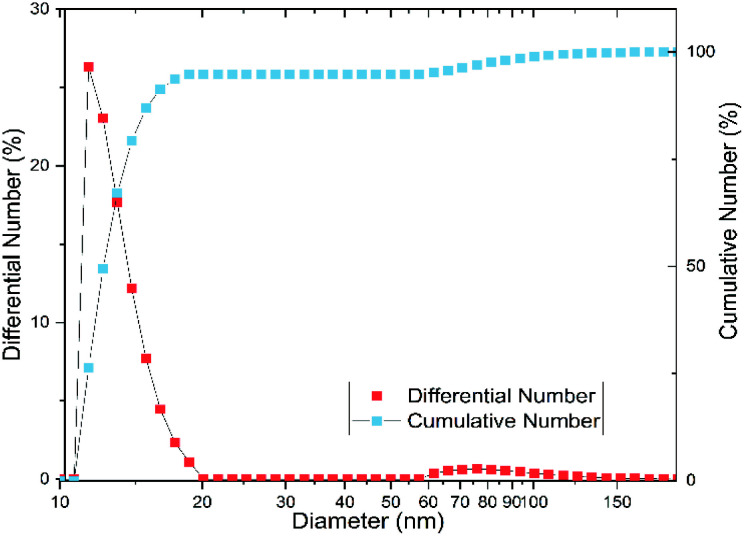
Particle size distribution measured by dynamic light scattering (DLS) results in a mean particle size of 11.7 ± 1.1 nm with a polydispersity index of 0.3 ± 0.1 (derived as the mean of three individual measurements). Red squares: number distribution, blue squares: cumulative number distribution.

In order to verify these effects on our substance, we examined the particle size and shape using dynamic light scattering (DLS), small-angle X-ray scattering (SAXS), and transmission electron microscopy (TEM). According to the DLS measurement, the particles have an average hydrodynamic diameter of 11.7 ± 1.1 nm, with most particles between 10 and 20 nm (*cf.*Fig. 3). The polydispersity index (PDI) is 0.3 ± 0.1, which corresponds to a moderately narrow size distribution.^31^ However, Fig. 3 shows a bimodal distribution with a fraction of aggregated particles, which comprises about 5% of the particles and is mostly between 60 and 150 nm.

**Fig. 4:**
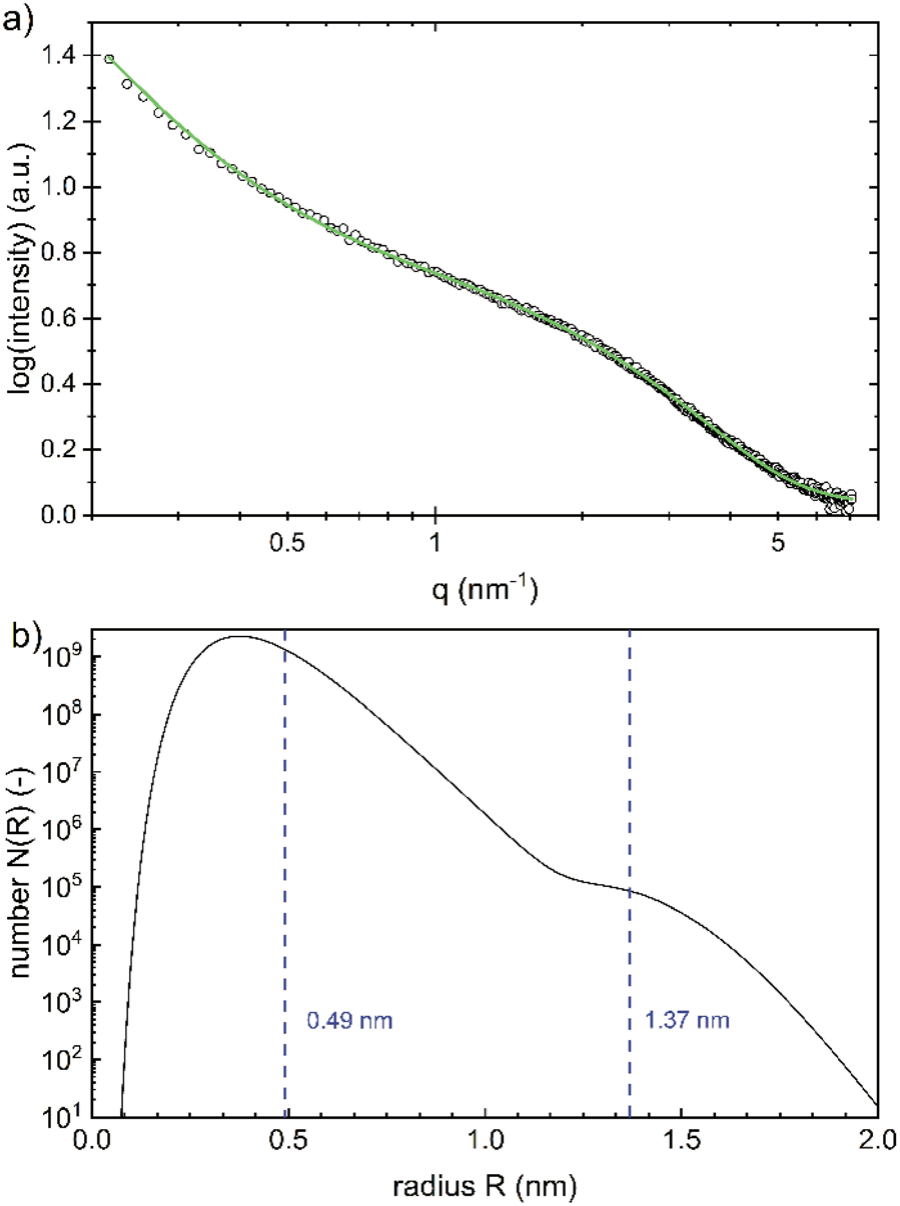
(a) SAXS data (black open circles) and the fitted spherical form factor (green line) according to (b) a bimodal log-normally distribution of the radius with median values of 0.49 ± 0.13 nm and 1.37 ± 0.14 nm, respectively. The errors are given as the standard deviation.

While the whole particles are recorded with DLS, the size of the iron(oxyhydr)oxide cores can be determined with SAXS, as the scattering length density (SLD) of iron(oxyhydr)oxide structures is larger than that of the organic components.^32^ The experimental result obtained by SAXS is shown in Fig. 4a.

The data were fitted using SASfit assuming a spherical form factor and an exponential background.^20^ As the SLD difference between the core nanoparticles and their coating is giving the strongest scattering contrast, we assume that the iron(oxyhydr)oxide core yields the main contribution to the scattering signal. No other scattering contributions were identified. A bimodal log-normal distribution of the radii of the spherical nanoparticles is observed. Fig. 4b shows the number distribution of particles on a logarithmic scale *versus* their radius. The majority of the nanoparticles have a very small radius of 0.49 ± 0.13 nm and only a small fraction is of larger size with 1.37 ± 0.14 nm. No structure factor is required for correct modeling of the measured SAXS data, as no regular order of the individual iron(oxyhydr)oxide nanoparticles could be found.

At a first glance, the results of DLS and SAXS appear very different. However, the iron(oxyhydr)oxide core structure is only recorded by SAXS whereas DLS records the hydrodynamic diameter of the particles in solution. Both, the SAXS and DLS measurements show bimodal distributions, but this is probably due to different causes. While particle aggregates are also determined using DLS, SAXS only records the size of the iron(oxyhydr) oxide cores within their organic shell. Therefore, we assume that the larger size fraction between 60 to 150 nm detected with DLS consists of loose nanoparticle aggregates. With DLS, a bimodal distribution of the main part of the particles with hydrodynamic diameters smaller than 20 nm cannot be determined due to the low resolution of the DLS technique.

The DLS and SAXS data show that the organic molecules bind to the surface of the iron(oxyhydr)oxide core and form an organic shell. Most stabilized particles have a diameter of approximately 11.7 nm with an iron(oxyhydr)oxide core diameter of less than 2.8 nm (calculated as double radius). As a result, a shell thickness between 3.6 to 5.9 nm can be calculated (calculation is shown in the ESI†). These ultrasmall sizes of the crystals are presumably the basis for the exceptional phosphate binding of this nanomaterial as reported by other authors.^9,10^ In general, the smaller the particle sizes, the larger their specific surface area and thus their adsorption capacity.^33^ Additionally, smaller particles generally show faster adsorption kinetics due to reduced diffusional resistance.^33^ Iron(oxyhydr)oxide cores smaller than 3 nm in diameter are very unusual. Other research studies report iron(oxyhydr)oxide cores of at least 6 nm (determined using SAXS).^32,34,35^ However, Weatherill *et al.* were able to demonstrate intermediate stages of Fe^3+^ polycations with a diameter of 0.9 nm, so-called Keggins, during their synthesis of 3 nm ferrihydrite nanoparticles.^36^ For these ultrasmall nuclei, the number of surface atoms is exceptionally large. For example, if the iron(oxyhydr)oxide structure was maghemite, a crystallite having a diameter of 2.8 nm would consist of approximately 3 unit cells in diameter with a cell dimension of 0.83474 nm as suggested by Cornell and Schwertmann.^15^ Assuming cubic crystallites, this corresponds to 38 unit cells with 302 iron ions of which 45 atoms are located on the surface, approximately 15% of the iron ions in the crystallite. In the case of crystallites with a particle diameter of 1.0 nm, however, 43% of the iron atoms would already be on the surface. Similar relationships result for other iron(oxyhydr)oxide structures as shown in Table S2 in the ESI.†

SAXS data indicate that the iron(oxyhydr)oxide cores are not regular in shape, which is also unusual. Although iron(oxyhydr)oxides can take various forms, they tend to have a regular shape, such as spheres, cubes, or rods.^15,37^ At the same time, other studies have shown that adding organic coating agents after the synthesis does not change the shape and size of the cores.^38^ In order to understand our results, the effects of organic components on nanoparticles have to be considered. There are two phases, in which the presence of organic matter can influence iron(oxyhydr)oxide nanoparticles. The first phase is during the formation of the nanoparticles. The second is after the precipitation of the particles and affects mainly the long-term stability of the nanoparticles. In general, the aqueous co-precipitation synthesis of iron(oxyhydr)oxides in a solution is based on spontaneous, homogeneous nucleation due to the supersaturation of the starting solution.^13^ This nucleation is followed by diffusional and coagulation growth.^13^ Organic substances that are already present during co-precipitation act as stabilizers of the nanoparticles and inhibit coagulation growth by binding on the surface *via* ligand exchange reactions, hydrogen bonding, and/or electrostatic attraction forces.^12,13^ While in our synthesis gum arabic was only dissolved shortly before drying, inulin and mannitol were present during the synthesis. For this reason, we suspect that inulin and mannitol influence the structure formation so strongly that larger, regular iron(oxyhydr)oxide nuclei cannot be formed. Both components are known for their stabilizing effect on iron(oxyhydr)oxide nanoparticle sizes.^26–28^ According to the authors' knowledge, the effect on the crystal structure has not been investigated for these substances yet. The combination of small molecules (mannitol) and longer polysaccharide chains (inulin) improves the protective function without steric hindrance between the coating molecules. In contrast to these two components, the gum arabic will only prevent particle aggregation after the synthesis. The particularly small size of the nanoparticles of this study increases the probability of aggregation, which in turn can lead to a reduction in phosphate binding.^33^ Due to the stabilizing effect, the high amount of organic material contributes to the exceptional phosphate binding of the nanomaterial. Assuming that the surface of the iron(oxyhydr)oxides is almost completely covered with inulin and mannitol, the gum arabic is expected to interact mainly with the hydroxyl groups of the inulin and mannitol molecules *via* amine or carboxylate groups. Similar effects are reported for the binding of gum arabic on aldehyde-functionalized magnetite nanoparticles.^39^

**Fig. 5:**
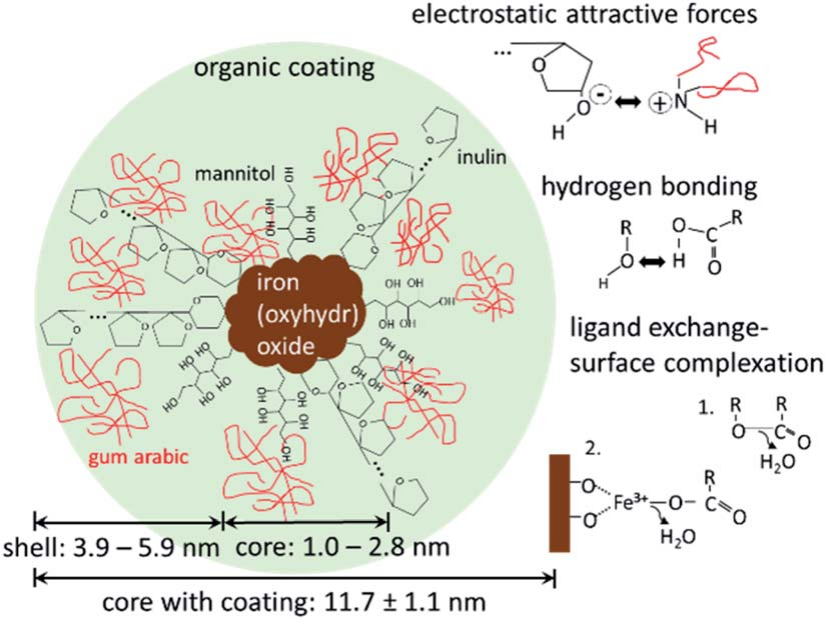
Schematic drawing of the active ingredient with iron(oxyhydr)oxide core and organic coating with mannitol, inulin, and gum arabic. Additionally, the main binding mechanisms of the organic molecules to iron(oxyhydr)oxide core as well as to other organic molecules are shown on the right side.

Fig. 5 shows a possible structure of the nanoparticles with the above-mentioned main binding mechanisms of the organic components on the iron(oxyhydr)oxide core surface and/or on other organic molecules.

**Fig. 6:**
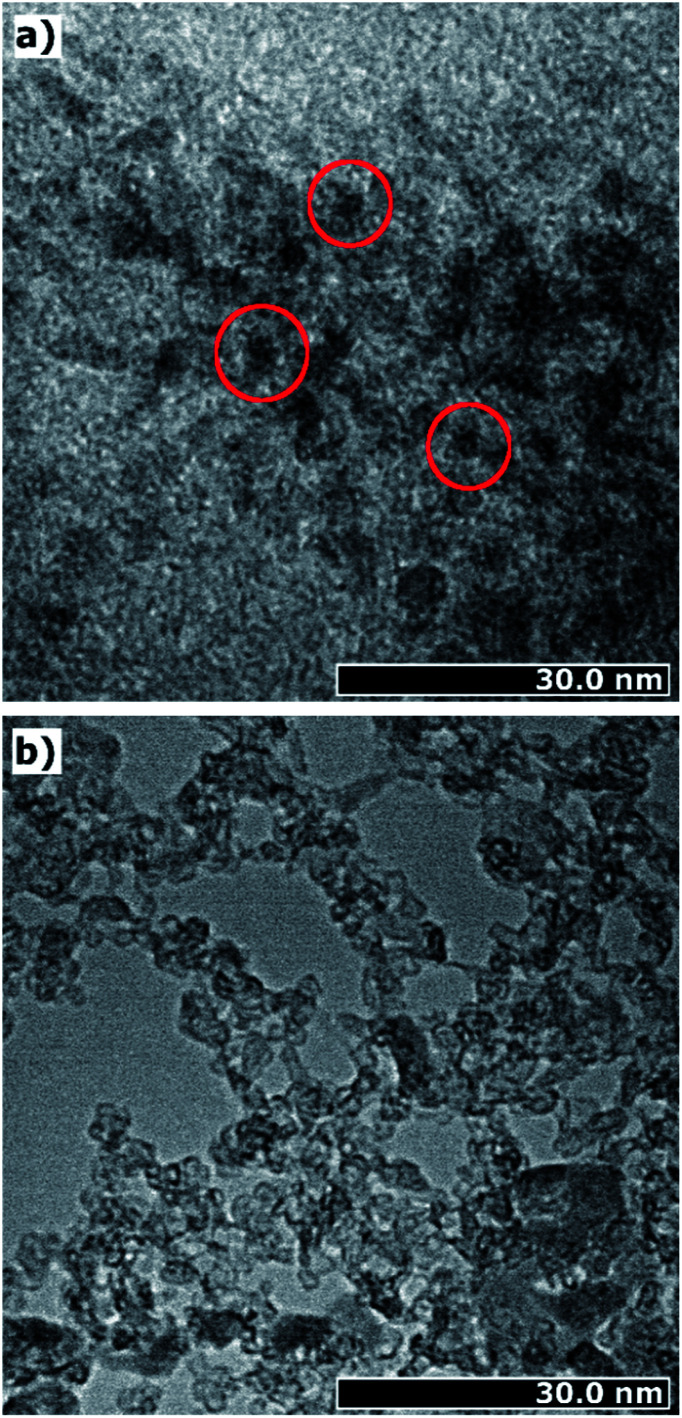
Transmission electron microscope (TEM) image of iron(oxyhydr)oxide-based nanoparticles: (a) accumulation of particles with iron(oxyhydr)oxide cores identifiable as dark spots, marked with red circles; (b) organic matter added as a coating material for the iron(oxyhydr)oxide nanoparticles.

In order to get a visual impression of the particle sizes and shapes, we made TEM images, which are presented in Fig. 6. The TEM images prove the presence of ultrasmall iron(oxyhydr)oxide cores, as shown as dark spots in Fig. 6a. No regular shapes are recognizable, in agreement with the SAXS data. In addition to the small nanoparticles, accumulations are observable due to aggregates (*cf.*Fig. 6a). However, these accumulations can easily be re-dispersed in water. This suggests that they are only held together in the dry state by weak physical forces, such as van-der-Waals forces, and may have been formed during drying.^25^ In addition to the dark spots, areas without crystalline structures can be identified as loose formations of organic substances (*cf.*Fig. 6b). In these areas, the organic molecules appear to form loose structures without being strongly bound to the core.

In addition to the particle size and shape, the high organic content is also likely to have an impact on crystallinity and crystal structure. The TEM images provide the first clue as they do not show a high contrast, which is known for weakly crystalline structures.^40^

In order to determine the degree of crystallinity and to identify the crystal structure in the iron(oxyhydr)oxide core, we performed an X-ray diffraction (XRD) measurement. However, the XRD data of the active ingredient of this study show no characteristic Bragg reflections (*cf.* Fig. S1 in the ESI†). Due to the lack of sensitivity of this method, small particle sizes and/or a low degree of crystallinity are known to lead to broadly smeared-out peaks and incomplete patterns that can hinder the identification of crystal structures.^18,41^ However, normally even very small iron(oxyhydr)oxide nanoparticles show a typical diffraction pattern, which has been observed, for example, for maghemite nanoparticles with diameters from 1 to 3.5 nm (ref. 42 and 43) as well as for ferrihydrite with a diameter of 1.6 nm.^44^ Thus, the absence of characteristic Bragg reflections in the XRD data has to result from a low degree of crystalline order of the iron(oxyhydr)oxide core.

**Fig. 7:**
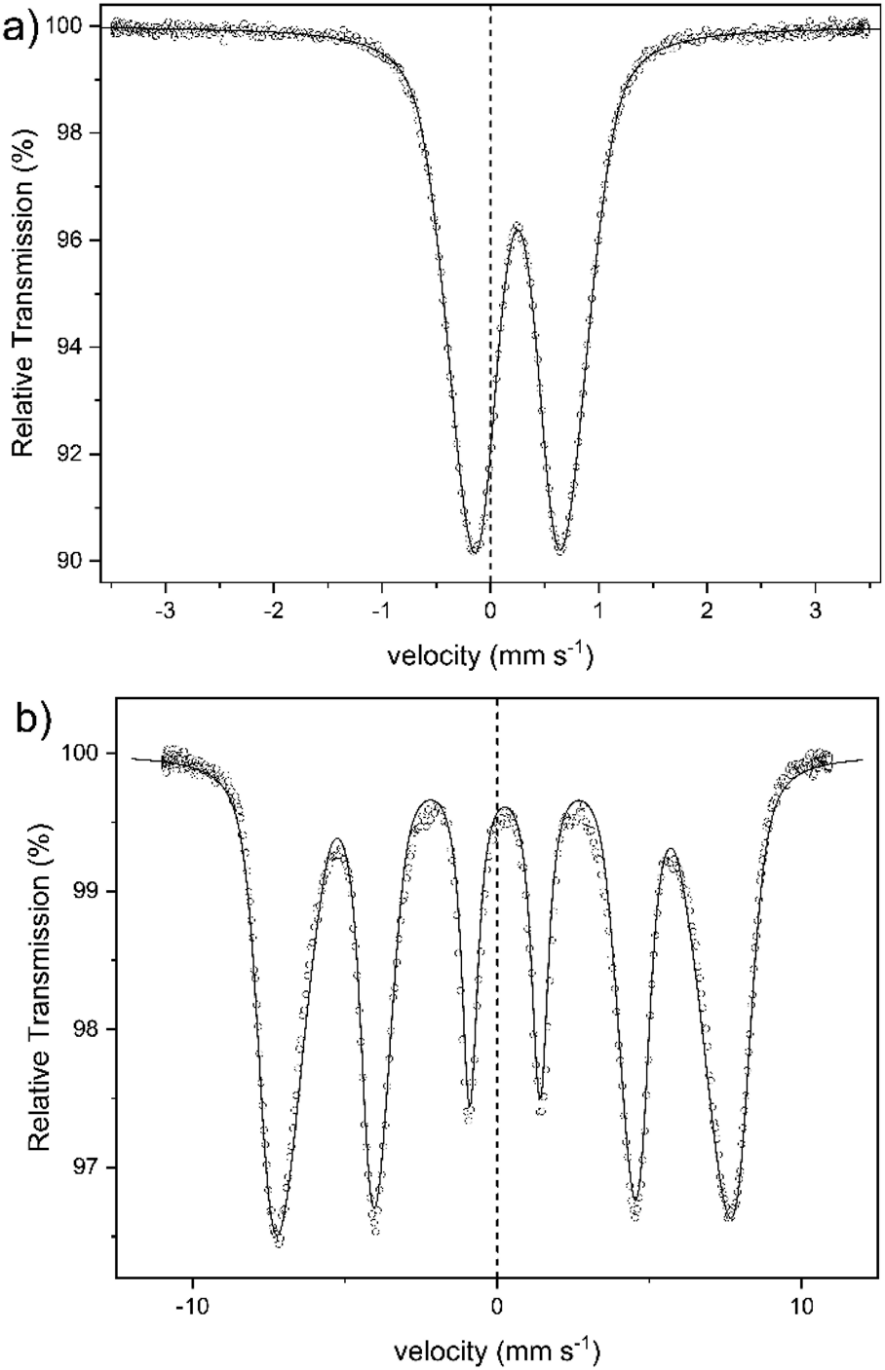
Mössbauer spectra of sample 1, obtained at (a) 300 K and (b) 4.2 K.

An alternative method for the examination of the nature of iron-containing particles is Mössbauer spectroscopy. Fig. 7 shows the spectra of sample 1 obtained at 300 K (a) and at 4.2 K (b). The other samples yielded almost identical spectra. The spectrum at 300 K shows a broad doublet, with an isomer shift of IS = 0.25(1) mm s^−1^, which is typical for the high spin of Fe^3+^-ions.^45,46^ There is no indication of the presence of Fe^2+^ in the spectra. This corresponds to the colorimetric analysis of the Fe^2+^ content in sample 1 (*cf.* Table S1 in the ESI†). The broad doublet can only be explained by a distribution of electric quadrupole splittings. Good fits could be obtained with an asymmetric Gaussian distribution of quadrupole splittings when the variance for splittings that are larger than the most probable one was assumed larger than that for splittings that are smaller than the most probable one. The fit shown in Fig. 7a was obtained in this way. The mean electric quadrupole splitting of QS = 0.85(1) mm s^−1^ agrees well with splittings previously obtained for ferrihydrite.^14,47–49^ Similar results have also been reported for maghemite,^46^ and lepidocrocite,^50^ although Murad and Schwertmann mentioned that room temperature quadrupole splitting is usually lower for iron(oxyhydr)oxides.^41^ However, several studies have demonstrated that the quadrupole splitting measured at 300 K increases with decreasing crystallite size^47^ and with decreasing degree of crystallinity.^14,41^ The 4.2 K spectrum (Fig. 7b) is a magnetic sextet with lines broadened by a distribution of hyperfine fields. It was fitted by assuming a Gaussian distribution of hyperfine fields with a variance that was allowed to be different for fields larger and smaller than the most probable one. The variance resulted larger on the low-field side and smaller on the high-field side. Such asymmetric distributions are usually observed for ferrihydrites.^24,41^ The mean hyperfine field obtained for sample 1 is 45.5(1) T. The most probable field is, because of the asymmetric distribution of hyperfine fields, 47.8(1) T. The reported hyperfine field at 4.2 K of most iron(oxyhydr)oxide is typically around 50 T or higher.^14,24,46,48,51–53^ For schwertmannite,^15,54^ lepidocrocite,^15,24,53^ goethit,^55^ and akaganéite^15,24^ as well as for ferrihydrite with a high organic matter content,^14,15^ however, lower hyperfine fields between 45 T and 48 T have been reported. However, the Mössbauer spectra are strongly influenced by the particle size and the degree of crystallinity.^14,56^ Therefore smaller particles show a lower hyperfine field at 4.2 K when the anisotropy energy of the particles is increased.^56^ For 6-line and 2-line ferrihydrite spectra evaluated in the same way, the mean and most probable fields do not differ much because the hyperfine field distribution is narrower, being around 49.5 and 50.5 T, respectively.^41^ If the structure of the present particles is similar to that of larger ferrihydrite particles, the lower fields may result in a larger proportion of iron ions near the surface of the particles, where the fields may tend to be lower than in the cores. A typical feature of low-temperature Mössbauer spectra of ferrihydrite is that nearly no electric quadrupole interaction is observable, although there is a substantial quadrupole splitting at room temperature. In magnetically split spectra observed at low temperature, the quadrupole interactions should result in a shift of the two outer lines with respect to the four inner lines. If the hyperfine field and the axis of the electric field gradient have the same direction, this quadrupole shift should be as large as the splitting in the room temperature spectra. In ferrihydrite, the angle between these two directions appears to vary more or less randomly, and the quadrupole shift in ferrihydrite is typically small and usually slightly negative with a magnitude below 0.1 mm s^−1^. For sample 1, the quadrupole splitting is QS = −0.03(1) mm s^−1^. The isomer shift at 4.2 K is practically the same as at room temperature.

In summary, the Mössbauer spectra of our samples can be explained by assuming that the cores are ferrihydrite particles smaller than those observed previously.^14,48,57,58^ The magnetic ordering temperature below which the particles become speromagnetic is known to increase with increasing particle size.^59,60^ The particles are superparamagnetic at room temperature but are blocked at low temperatures and then show the magnetic hyperfine spectra. Therefore, we cannot definitely identify any crystal structure, but the results indicate a low crystalline ferrihydrite as the main crystal structure of the active ingredient. Furthermore, other iron(iii)-containing structures could not be definitively excluded, as their assignement is complicated by effects due to the ultrasmall particle size.

**Fig. 8:**
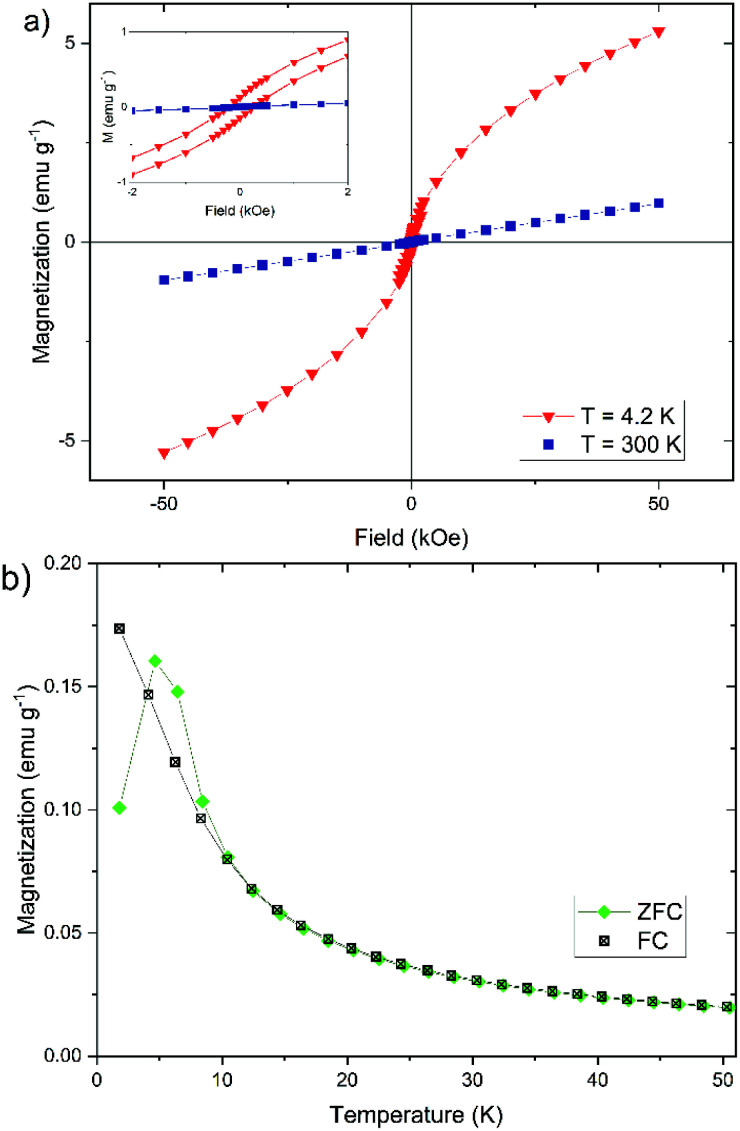
Magnetization measurements of synthesized iron(oxyhydr)oxide-based nanoparticles for sample 1: (a) dependence of magnetization M on external magnetic field from −50 kOe to 50 kOe at 300 K and 4.2 K and (b) field-cooled (FC) and zero-field-cooled (ZFC) magnetization curves under an external field of 0.2 kOe.

In addition to the Mössbauer spectra, the magnetic properties can also offer further information about the crystal structure, the degree of crystallinity, and the core size. For this reason, we have determined the dependence of the magnetization of our nanoparticles on the strength of an external magnetic field (*cf.*Fig. 8a) and on temperature (*cf.*Fig. 8b). The magnetization of the nanoparticles at 300 K changes linear with the external field (*cf.*Fig. 8a), which is typically for a paramagnetic behavior.^61^ This observation agrees well with the doublet measured by Mössbauer spectroscopy in Fig. 7. The magnetization curve at 4.2 K has a sigmoidal shape with an almost negligible coercivity of 0.24 kOe and a remanence magnetization of 0.13 emu g^−1^. It is not saturated up to an external magnetic field of 50 kOe, which is a typical behavior observed for superparamagnetic materials.^61^

In general, the magnetization behavior strongly depends on the crystal structure.^47,62^Table 1 gives an overview of the type of magnetism to which the different iron(oxyhydr)oxide structures are assigned. Basically, nanoparticles of different structures can be divided into two groups: the ferrimagnetic materials and the substances exhibiting antiferromagnetism (or in the case of ferrihydrite speromagnetism, a special spin arrangement resembling random antiferromagnetism).^63^ The iron(oxyhydr)oxides with ferrimagnetic behavior like maghemite, magnetite, ε-Fe_2_O_3_, and feroxyhyte typically show hysteresis curves with remanent magnetization and coercivity at room as well as at cryogenic temperatures.^46^ Depending on the temperature and particle size, these substances reach high saturation magnetizations.^46,47,62^ A lowering of the temperature leads to an increase of the saturation magnetization since the thermal fluctuations of the magnetic spins on the nanoparticle surface are reduced.^62^ The change in the composition or the size of the magnetic nanoparticles can cause a change in the magnetic properties. It has been shown that the exchange of individual iron atoms in magnetite with other atoms greatly reduces the saturation magnetization.^64^ A decrease in the saturation magnetization with decreasing particle sizes and/or decreasing degree of crystallinity was shown for maghemite,^65,66^ magnetite,^67,68^ and feroxyhyte.^69^ This is influenced by the increase of the proportion of surface atoms due to the spin canting, *i.e.* a lack of complete alignment of the magnetic spins of surface atoms.^64,66^ In addition, the magnetization can change the direction randomly induced by thermal excitation in the case of small ferromagnetic nanoparticles. In the absence of an external magnetic field, the time between these flips of the magnetization can be shorter than the measurement time for recording the magnetization. As a result, the average magnetization appears to be zero, as with paramagnetic materials, and no remanent magnetization or coercivity is detectable. This state is called superparamagnetism.^70^ Therefore, superparamagnetic nanoparticles show a sigmoidal curve at room temperature and at 4.2 K the nanoparticles exhibit hysteresis with remanent magnetization and coercivity as the magnetic moments of the particles are blocked.^62^ Although the particle size and degree of crystallinity have a large influence on the magnetization, it has been shown that ferrimagnetic materials with ultrasmall particle sizes probably still exhibit the sigmoidal shape at room temperature. Examples are maghemite nanoparticles with a diameter of 1.5 nm and 3 nm.^64,66,71^ This is in contrast to the observed linear behavior shown in Fig. 8a. Antiferromagnetic materials show paramagnetic behavior at room temperature with linear dependence of the magnetization on the external magnetic field as shown for akaganéite^72,73^ and lepidocrocite.^74^ At cryogenic temperatures, these materials can show hysteresis as uncompensated magnetic spins on the surface contribute to the magnetic behavior.^47^

**Table 1:** Overview of the most common iron(oxyhydr)oxide crystal structures with respect to structural formulae and type of magnetism. Accordingly, the authors provide an assessment if the investigated properties of the nanoparticles of this study are in accordance to the properties with the specific iron(oxyhydr)oxide

Containing Fe^2+^ and/or Fe^3+^?	Containing oxide and/or hydroxide?	Substance name	Structural formulae	Type of magnetism	Are the properties of nanoparticles of this study in accordance with the properties of the structure?
Fe^2+^	Iron oxide	Wüstite	FeO	Antiferromagnetic^15^	No, no Fe^2+^ contained
	Iron hydroxide	Iron(ii)hydroxide	Fe(OH)_2_	Antiferromagnetic^82^	No, no Fe^2+^ contained
Fe^2+^ and Fe^3+^	Iron oxide	Magnetite	Fe_3_O_4_	Ferrimagnetic^15^	No, no Fe^2+^ contained, differing magnetic properties, and differing Mössbauer spectra
Fe^3+^	Iron oxide	Hematite	α-Fe_2_O_3_	Canted antiferromagnetic^83^	No, differing hyperfine field at 4.2 K (ref. 84)
		Iron oxide beta phase	β-Fe_2_O_3_	Antiferromagnetic^85^	Yes, but the structure is thermodynamically unstable, which is in contrast to the nanoparticles of this study^81^
		Maghemite	γ-Fe_2_O_3_	Ferrimagnetic^62^	No, differing magnetic properties
		Iron oxide delta phase	δ-Fe_2_O_3_	Ferrimagnetic^86^	No, differing magnetic properties
		Iron oxide epsilon phase	ε-Fe_2_O_3_	Ferrimagnetic^87^	No, differing magnetic properties
	Iron oxyhydroxide	Goethite	α-FeOOH	Antiferromagnetic^37^	No, differeing Mössbauer spectra^55^
		Akaganéite	β-FeOOH	Antiferromagnetic^88^	No, hardly any chloride-ions contained
		Lepidocrocite	γ-FeOOH	Antiferromagnetic^15^	Yes
		Feroxyhyte	δ′-FeOOH	Ferrimagnetic^15^	No, differing magnetic properties and higher hyperfine field at 4.2 K (ref. 84)
		Ferrihydrite	Fe_5_HO_8_·4H_2_O	Speromagnetic^15^	Yes
		Schwertmannite	Fe_8_O_8_(OH)_6_(SO_4_)·*n*H_2_O	Antiferromagnetic^89^	No, no sulfate contained
	Iron hydroxide	Bernalite	Fe(OH)_3_	Antiferromagnetic^90^	No, differing hyperfine field at 4.2 K (ref. 84)

Overall, the nanoparticles show magnetic properties that differ from ferrimagnetic substances, since the magnetization changes linearly with the external field at room temperature. We assume that the ultrasmall size of ferrimagnetic crystallites does not lead to this effect. To the authors' knowledge, there is neither a confirmatory nor a refuting study in the literature. For this reason, we assume that an antiferromagnetic or speromagnetic iron(oxyhydr)oxide is prevalent in the nanoparticle core, but cannot finally exclude ferrimagnetic structures.

The temperature dependence of the magnetization can also provide important information about the magnetic properties of the nanoparticles (*cf.*Fig. 8b). The field-cooled (FC) magnetization steadily decreases from the lowest temperature to the highest temperature. The zero-field-cooled (ZFC) magnetization shows a characteristic increase between 2 K and 4.7 K, the so-called blocking temperature *T*_B_. Above 4.7 K the curve behaves largely like in the FC case. In the temperature range between 4.7 K and 10 K, the ZFC magnetization lies above the FC magnetization. The curves show a behavior that is almost typical for the magnetization of iron(oxyhydr)oxide nanoparticles.^46,47,52,62,75,76^ It is, however, unusual that the ZFC magnetization is partly higher than the FC magnetization between 4.7 K and 10 K.

The same effect was observed by Kollu *et al.* for a nano compound made of reduced graphene oxide-nickel and nickel ferrite.^77^ This atypical behavior was evident when recording the ZFC and FC magnetization at low magnetic fields (100 and 200 Oe). The phenomenon disappeared at higher magnetic fields (500 and 2000 Oe) and the curves behaved as expected. The researchers explained this by a competition between ferromagnetic and antiferromagnetic regions in the material. If the external magnetic field is sufficiently small, these interactions can lead to unpaired spin systems, which lead to this anomalous behavior of the temperature-dependent magnetization curves.^77^

In addition, the measured blocking temperature is extremely low compared to other iron(oxyhydr)oxide nanoparticles.^47,52,62,76^ However, it should be noted that the blocking temperature depends on the crystal size as well as on the distance between the crystallites.^46,60,78,79^ The larger the crystals, the higher the blocking temperature.^60,79^ Due to the polydispersity of the particles, there are several different blocking temperatures, the superposition of which creates the maximum in the ZFC magnetization behavior. Consequently, the width of the particle size distribution can also be inferred from the peak width in the ZFC curve.^46,79^ In addition, the blocking temperature also decreases with increasing distance between the crystallites, since the interactions are decreased, which reduces the formation of magnetic moments, like observed by Berquó *et al.*^78^ These authors lowered the blocking temperatures of their maghemite nanoparticles from 50 K to 12 K by coating the particles with alginate and sugar.^78^ To our knowledge, however, no such low blocking temperatures have been described for iron(oxyhydr)oxide nanoparticles in the literature. For example, other authors reported a blocking temperature of 60 K for ferrihydrite nanoparticles with a size of 1.6 nm or 29 K for measuring maghemite nanoparticles with a size of 2.5 nm.^47,80^ We, therefore, believe that the ultrasmall particle size of our iron(oxyhydr)oxide cores alone does not cause this low blocking temperature. Only the combination with a large amount of organic material, which causes a large distance between the particles even in the dried state, leads to this extreme reduction of *T*_B_.

Overall, like with the XRD and the Mössbauer spectra, it is not possible to finally identify a crystal structure based on the magnetization curves, as the influences of crystal structure, degree of crystallinity, and particle size complicate a structural classification. However, all methods provide hints, which we summarize in Table 1. The active ingredient does not contain Fe^2+^-ions or sulfate as well as hardly any chloride ions. Therefore, we can exclude schwertmannite, akaganéite, and all Fe^2+^-containing iron(oxyhydr)oxides. As mentioned above, many iron(oxyhydr)oxides, like maghemite, hematite, and feroxyhyte normally show a larger magnetic hyperfine field at 4.2 K. Based on the SQUID measurements, we suspect additionally a para- or speromagnetic behavior of the structure. β-Fe_2_O_3_ is known to be paramagnetic at room temperature and antiferromagnetic at 4.2 K, but it is thermodynamically unstable and transforms to maghemite or hematite.^81^ Since our experience with this nanomaterial shows that the crystal structure is stable, we also exclude a structure based on β-Fe_2_O_3_. Final identification of the structure is not possible due to the ultrasmall particle size and we cannot exclude that ultrasmall iron oxides like maghemite do not show similar properties. Nevertheless, we conclude that the nanoparticles are probably low-crystalline ferrihydrite or, less likely, lepidocrocite.

## Conclusions

We investigated the structure and physical properties of a novel active ingredient for hyperphosphatemia treatment.

The substance synthesized by co-precipitation is based on iron(oxyhydr)oxide nanoparticles with a shell of organic substances. Therefore, inulin and mannitol are added during the synthesis, wherefore they interact with the crystal surfaces during the formation of the iron(oxyhydr)oxide crystallites. As a result, coagulation growth is suppressed and ultrasmall iron(oxyhydr)oxide crystals with diameters between 1.0 to 2.8 nm are formed. After the synthesis, gum arabic is additionally added. During storage, all three organic substances prevent particle aggregation. The nanoparticles with the coating have an average diameter of approximately 11.7 nm. In addition to the particle size, the organic substances influence the formation of crystal structures and the degree of crystallinity. However, the structural classification of the iron(oxyhydr)oxide core was complicated due to the influences of the degree of crystallinity and particle size. We were able to exclude schwertmannite, akaganéite, and all Fe^2+^-containing structures on basis of our compositional analysis. The Mössbauer spectrum measured at 4.2 K showed a low hyperfine field of 45.5 T, which is unusual for iron(oxyhydr)oxides. Similar results could only be achieved with low crystalline nanoparticles with larger amounts of organic components. We demonstrated that the material shows no typical ferrimagnetic properties. Overall, a crystal structure could not be fully identified. Due to our results, we conclude that the particles are low crystalline ferrihydrite or, less probably, lepidocrocite. Final identification of the structure is not possible due to the ultrasmall particle size and we cannot exclude that ultrasmall iron oxides like maghemite do not show similar properties.

However, our results show that the active ingredient, due to its special mixed composition, has extraordinary properties like ultrasmall crystal sizes with an extremely large number of surface atoms. The exact binding mechanisms of phosphate to the active ingredient should be further investigated in a separate study. Interesting aspects are generally the binding capacity and the adsorption kinetic. The identification of possible binding mechanisms, such as pure adsorption of the phosphate on the iron(oxyhydr)oxide surface or the incorporation of the organic shell is also an interesting research topic. With our investigations, we have created the basis for these research studies.

Moreover, we have shown in this study an approach to a combinatorial characterization of nanomaterials as proposed by Mourdikoudis *et al.*^18^ Ultrasmall nanoparticles can have very special properties. At the same time, however, the investigation of these particles is rendered, if not impeded, by their small size. Nevertheless, nanoparticles with sizes smaller than 3 nm are used in many disciplines, such as in nanomedicine or electrochemistry.^91,92^ In this study, we presented an approach to investigate the structure and shape of ultrasmall nanoparticles by the simultaneous application of chemical analysis and a variety of physical methods. Our approach can help in future studies to examine the structural properties of a wide variety of nanoparticles with ultrasmall particle sizes.

## Conflicts of interest

There are no conflicts to declare.

## Supplementary Material

RA-011-D1RA00050K-s001
