# Supplementary material for: Characterization of an active ingredient made of nanoscale iron(oxyhydr)oxide for the treatment of hyperphosphatemia
Source: RSC Adv. 2021 May 14;11(29):17669–82. doi: 10.1039/d1ra00050k (PMC9033185; doi:10.1039/d1ra00050k)
Supplement: RA-011-D1RA00050K-s001 [file RA-011-D1RA00050K-s001.pdf]

Electronic Supplementary Material (ESI) for RSC Advances.
This journal is © The Royal Society of Chemistry 2021

Electronic Supplementary Information (ESI)

Characterization of an active ingredient made of nanoscale iron(oxyhydr)oxide for the treatment of hyperphosphatemia

Received 00th January 20xx,
Accepted 00th January 20xx

DOI: 10.1039/x0xx00000x

Magdalena Bäumler[ab], Sebastian P. Schwaminger[a], Daniela von der Haar-Leistl[b], Simon J. Schaper[c], Peter Müller-Buschbaum[cd], Friedrich E. Wagner[e], and Sonja Berensmeier*[a]

Table S1 Composition of iron oxide based nanoparticles used in this study: mass fractions ω (mg g^{-1}) of the main components iron, Fe^{2+}-ions, inulin, mannitol and gum arabic as well as concentration of the residual contained secondary components sodium and chloride. Due to the small production scale and as a test for reproducibility, we prepared three samples named sample 1 to 3.

	ω (Fe) (mg g^{-1})	ω (Fe^{2+}) (mg g^{-1})	ω (inulin) (mg g^{-1})	ω (mannitol) (mg g^{-1})	ω (gum arabic) (mg g^{-1})	ω (Na^{+}) (mg g^{-1})	ω (Cl^{-}) (mg g^{-1})
sample1	210.06 ± 3.45	1.78 ± 0.18	146.30 ± 9.73	106.40 ± 5.69	338.09 ± 19.50	4.32 ± 0.16	0.49 ± 0.08
sample 2	220.58 ± 1.57	1.32 ± 0.12	154.26 ± 9.93	124.81 ± 5.24	332.21 ± 22.37	3.64 ± 0.06	0.49 ± 0.09
sample 3	228.62 ± 6.87	3.14 ± 0.37	137.14 ± 9.27	120.14 ± 6.99	317.53 ± 40.43	4.42 ± 0.12	0.40 ± 0.12

Calculation of mean organic shell thickness:

Results from SAXS-data:

Mean radius of iron(oxyhydr)oxide core r_{core} = 0.49 to 1.37 nm

=> diameter of core $d_{core} = 2^* r_{core}$ = 0.98 to 2.74 nm

Results from DLS-data:

Mean diameter of nanoparticles $d_{particle}$ = 11.7 ± 1.1 nm = 10.6 to 12.8 nm

⇨ Minimal shell thickness $t_{shell, minimal} = 0.5^*(d_{particle, minimal} - d_{core,maximal})$=0.5*(10.6 nm – 2.74 nm) = 3.93 nm

⇨ Maximal shell thickness $t_{shell, maximal} = 0.5^*(d_{particle, maximal} - d_{core,minimal})$=0.5*(12.8 nm – 0.98 nm) = 5.91 nm

a. *Bioseparation Engineering Group, Department of Mechanical Engineering, Technical University of Munich, Boltzmannstraße 15, 85748, Garching, Germany E-mail: s.berensmeier@tum.de*
b. *Fraunhofer Institute for Process Engineering and Packaging (IVV), Department of Process Development for Plant Raw Materials, Giggenhauser Str. 35, 85354 Freising, Germany*
c. *Functional Materials Group, Departement of Physics, Technical University of Munich, James-Franck-Straße 1, 85748 Garching, Germany*
d. *Heinz Maier-Leibnitz Zentrum (MLZ), Technical University of Munich, Lichtenbergstr. 1, 85748 Garching, Germany*
e. *Experimental Astro-Particle Physics Group, Departement of Physics, Technical University of Munich, James-Franck-Straße 1, 85748 Garching, Germany*

Please do not adjust margins

Table S2 Total number of iron ions and number of surface iron ions as well as their distribution within crystallites having a diameter of a) 2.8 nm and b) 0.98 nm for different iron(oxyhdyr)oxide structures. Information on the crystal systems, cell dimensions and the formula per unit were extracted of literature[14]

**Oxide**	**lepidocrocite**	**akaganéite**	**ferrihydrite**	**hematite**	**maghemite**	**feroxyhyt**	**goethite**	**magnetite**
Crystal system	Ortho-rhombic	monoclinic	hexagonal	rhombohedral hexagonal	cubic	hexagonal	ortho-rhombic	cubic
Cell dimension a (nm)	0.3070	1.0546	0.2955	0.5036	0.8347	0.2930	0.9956	0.8396
Cell dimension b (nm)	1.2530	0.3031	0.2955	0.5036	0.8347	0.2930	0.3022	0.8396
Cell dimension c (nm)	0.3880	1.0483	0.9370	1.3749	0.8347	0.4560	0.4608	0.8396
Formula units per cell	4	8	4	8	8	2	4	8
iron ions per side	0.67	0.67	0.67	0.67	0.67	0.67	0.67	0.67
**a) crystallite diameter: 2.8 nm**								
number of unit cells in crystallite	147	66	268	63	38	561	158	37
number of iron ions in crystallite	588	524	1073	504	302	1122	633	297
number of iron ions on surface	136	75	195	72	45	280	133	45
proportion of surface iron ions	23%	14%	18%	14%	15%	25%	21%	15%
**b) crystallite diameter: 0.98 nm**								
number of unit cells in crystallite	6	3	12	3	2	24	7	2
number of iron ions in crystallite	25	22	46	22	13	48	27	13
number of iron ions on surface	17	9	24	9	6	34	16	5
proportion of surface iron ions	66%	41%	52%	41%	43%	71%	60%	43%

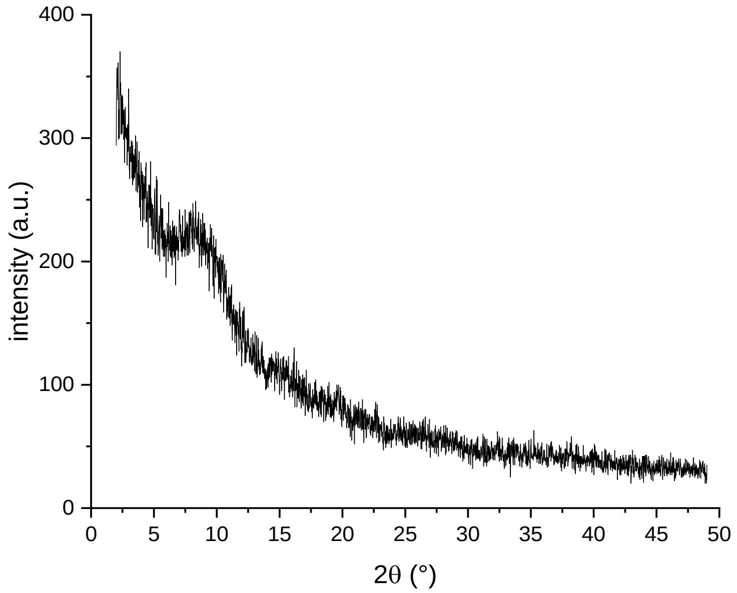

Figure S1 XRD data of sample 1

This journal is © The Royal Society of Chemistry 20xx

Please do not adjust margins
